# Familial Characteristics of Street Children in Tehran, Iran

**Published:** 2014

**Authors:** Ahmad Reza Ahmad Khaniha, Mitra Hakim Shooshtari, Mehrdad Mohammadian, Reza Bidaki, Ahmad Pourrashidi Boshrabadi

**Affiliations:** 1 Associate Professor, Mental Health Research Center, Tehran Institute of Psychiatry, School of Behavioral Sciences and Mental Health, Iran University of Medical Sciences, Tehran, Iran.; 2 Assistant Professor, Mental Health Research Center, Tehran Institute of Psychiatry, School of Behavioral Sciences and Mental Health, Iran University of Medical Sciences, Tehran, Iran.; 3 Assistant Professor, Department of Psychiatry, School of Medicine, Rafsanjan University of Medical Sciences, Rafsanjan, Iran.; 4 Medical Student, Department of Psychiatry, School of Medicine, Rafsanjan University of Medical Sciences, Rafsanjan, Iran.

**Keywords:** amilial Characteristics, Iran, Street Children, Tehran

## Abstract

**Objective:** The phenomenon of street children is one of the most important concerns facing global community. Identifying risk factors in such children could lead to crucial investigations to find their essential needs by intervention programs. The present study examined the family profile of street children in Tehran, Iran.

**Methods:** The sample consisted of 576 street children who were evaluated by 15 examiners. Using multi-stage sampling, twenty-seven different areas of Tehran were divided into 5 clusters and the children were selected randomly from each cluster. The two questionnaires applied included a demographic questionnaire and a questionnaire about high-risk behaviors such as substance use, alcohol consumption, cigarette smoking, etc.

**Results: **There was a statistical significant association between the length of work time and two variables: sex and economic status of family (p < 0.05). Incarceration history of street children who had lost both their parents and their parents had been divorced was significantly higher than others (p < 0.05).

**Conclusion:** The street children's lifestyle is a risk factor for affecting them to variety types of socio-mental problems. Statistical significant association between parental divorces or the loss of both parents with a history of conviction mentions the important role of parents in transmitting moral and social values to children.

## Introduction

During recent decades, facing with irregular and troubled children in a busy street has attracted the community toward this problem. At first view, this problem is insoluble, and these children are considered lost. But all these children do not have the same problem (1). It seems that one of the most important concerns facing global community is the unclear estimation for the number of these children (2). Determination the number of these children is difficult due to the difference in statistics or a lack of consensus on the definition of such children (3). Other causes of differences in the statistics include their continuous migration and lack of identity cards or birth certificates (4).

United Nations International Children's Emergency Fund** (**UNICEF) estimates that 100 million of such children live throughout the world (3), but some of the statistics have estimated this number to be 150 million (5, 6). According to Andres-Lemay et al., street children were mainly of atypical families and 78% of them had single parents or no parents at all. Furthermore, in UNICEF study in Ethiopia, 23% of street children lived with both parents and in Brown's study in Jamaica, 7% lived with their both parents (7).

There are different definitions of street children by the World Health Organization (WHO), UNICEF and other agencies. UNICEF has divided them into three groups: 1. Children who are away from family and live on streets; 2. Children who work on streets, spend a lot of time on streets, but come back to home regularly; and 3. Children who live with their families on streets.

Various factors such as economic pressures, unequal distribution of wealth, lack of social services, AIDS and civil wars are effective in the incidence of the phenomenon of street children (5). Other effective factors also include discrimination, abuse, family violence, drug use, loss of parents, low parental education, and immigration (4, 5). 

There is little information about demographic and family background of such children in Iran. Although we do not have access to exact information of the severity and extent of street children in Iran, but previous statistics show 200,000 street children currently settle in Iran (8, 9).

Finding the precise characteristics of these children is the first step in helping them. This study was conducted with objective of addressing this issue.

## Materials and Methods

This study was conducted in Tehran (the capital of Iran). Using multi-stage sampling, twenty-seven municipality districts of Tehran were divided into 5 clusters. A total of 576 street children were selected randomly from each cluster in February 2008. Street children in this study refer to children 10 to 19 years who spend days and nights on streets with or without family. 

The 15 interviewers were trained how to fill the questionnaires. The checklists used were designed by the researchers and included 30 questions. A Pearson coefficient of 0.08 calculated showed good interrater reliability.

At first, the interviewers took consent form children by explaining the goal of the study and emphasizing on confidentiality of their outcomes. They conducted their work under comments of a supervisor. Interviewers were students of psychology course. The questionnaires were filled in by interviewers included a demographic questionnaire, and a questionnaire about substance abuse. There were some questions about the cause of being street children in the questionnaire. All children interviewed signed consent form. 

All data collection then was analyzed using the SPSS for Windows 17.0 (SPSS Inc., Chicago, IL, USA). Statistical significance was estimated by the Chi-square and Fisher's exact test. Significance level was defined as p < 0.05.

## Results

A total of 579 street children were interviewed in this study. There were 494 (84.2%) males and 90 (8.15%) females. Mean (±SD) age of males was 13.7 (±4.7) years and of females was girls were 11.1 (±2.3) years (p < 0.05). The educational status of 36.8% of fathers and 42.2% of mothers were illiterate. About 79.7% of fathers were self-employed and 64.4% of mothers were housewives. About 10% of street children had stepmother and 5% had stepfather. Regarding living place, 47.7% of the interviewed street children lived with their grandfather and/or grandmother. Half of them mentioned that father was the only source of household income and 31% mentioned sister or brother as the source of family income. 

About 61.8% of street children were working throughout the year constantly. The length of working time of the street children was associated with two variables of sex and economic status of the family (p < 0.05); 63.4% of street boys worked throughout the year and 87% of children who constantly worked over the year were boys and this difference is quite significant with girls (p < 0.05).

The 77% of children, who worked during the year, permanently had lower levels of education or guidance and 20% had a history of dropout. The children who worked during the year constantly most were in the low economic level (p < 0.05). The 76% of children handed their earnings to the families.

The 36.4% of children had smoked cigarette in their lifetime. Prevalence of smoking in boys were 38.7% which was significantly higher than in girls (23.4%); p < 0.05. Mean (±SD) number of smoked cigarettes in girls 12.1 (±0.1) was significantly lower than in boys 14.5 (±2.7).

The 7.8% of children had a history of alcohol consumption and significant differences between sexes were observed in the relative frequency of alcohol use. The average age of alcohol consumption was significantly lower in girls than in boys. About 3.6% of children reported other illicit drugs use. There was no statistical significant difference between the girls and boys regarding this behavior (Table 1).

The average age of girls using drugs was significantly lower than the mean age of boys (Table 2).

47 (8.3%) street children had a history of conviction. In other words, 9% of males and 4.6% of female population had a history of criminal conviction. The significant difference between groups was found in conviction record. The mean age of the boys with a history of incarceration was 13.8 and in girls was 12.7 years. However, 15% of children mentioned illegal behaviors. History of incarceration the street children who had lost both their parents or separated parents was significantly higher than the other street children (p < 0.05).

## Discussion

Collecting data about family characteristics of street children can be the first step for any intervention. The results showed that almost half of the street children lived with their grandparents. It shows the important role of support presenting by Iranian extended family. Raffaelli showed that family disruption is an important factor in the street youth phenomenon, and having a place for living might be more important than poverty (10).

Probably, due to the economic problems in these families, children are forced to work on streets. In a research that has examined the situation of street children in Rwanda, it was found that half of homeless children were orphaned or lived with one parent. Factors such as death of family members, being imprisoned and poverty have effective role in creating street children (11). Based on the current findings, 40% of their parents were illiterate. The majority of such parents in Pakistan also were illiterate regardless to the employment status (11). In the present study, in spite of being fathers as the main source of income for the family in about 50% of children, 76% of children gave their income to the family members. It accounts a type of emotional abuse and it is against children's rights.

The low educational level, lack of occupational skill, economic problems, and unemployment, all has negative roles in life of street children. About 8.3% of the studied street children had a history of incarceration and antisocial behavior was found in 15% of cases. The findings of this study showed that age of illegal drug use was significantly lower in girls than in boys. There are not similar findings in other studies. Lower age of illicit drug abuse in girls make them more vulnerable to adverse environmental conditions that may increase psychopathology. The lower age of girls being involved can probably get them into the substance as a source of income. In many cases, vendors use street girls for transportation of drugs. It is consistent with other studies. A study conducted in developing countries reported that homeless girls are more likely to be from dysfunctional families and exhibit psychological distress than their male peers (10, 11). Then they have reported that, street girls had poorer outcome relative to boys because of disruption of social norms (12, 13).

**Table 1:** Comparison of drug use between male and female street children in Tehran, Iran

**Variable**	**n (%)**	**n (%)**	**Significant level**
**Cigarette **	189 (38.7%)	21 (23.4 %)	p < 0.05
**Alcohol **	220 (45%)	8 (8.8%)	p < 0.05
**Other drugs**	5 (1%)	1 (1.1%)	p > 0.05
**Total use of substance**	489 (84.4%)	90 (15.5%)	p < 0.05

**Table2:** Age of drugs/alcohol initiation in street children in Tehran, Iran

**Variables**	**Males**	**Females**	**Significant level**
**Cigarette**	14.5 (±2.7)	12.1 (±2)	p < 0.05
**Alcohol **	14.9 (±2.3)	12.5 (±2.5)	p < 0.05
**Stimulants **	13	15	p < 0.05
**Any substance**	13.7 (±4.7)	11.1 (±2.1)	p < 0.05

Data are shown in Mean (±SD).

In the study of Nigeria, conviction history because of street violence and drug use were observed in 1.3% of street children (14). It is also important to note that although the number of girls was less than the boys, the rate of antisocial behavior was higher in girls compared to boys. 

Significant association between parental divorces or the loss of both parents with a history of conviction mentions the important role of parents in transmitting moral and social values to children. Given the matters listed the life pattern of street children make them vulnerable to psychosocial problems. Recognition of risk factors about street children and their families can lead to identification of a series of principles to address the needs of intervention to decline their problems.

## Authors' contributions

ARAKh conceived and designed the evaluation and helped to draft the manuscript. MHSh participated in designing the evaluation and performed parts of the statistical analysis. MM participated in designing. RB and APB re-evaluated the clinical data, performed the statistical analysis and revised the manuscript. All authors read and approved the final manuscript.

## Footenote

**Declaration of interest:** None.
